# A Phase 3, Double-Blind, Randomized, Active Controlled Study to Evaluate the Safety of MenAfriVac in Healthy Malians

**DOI:** 10.1093/cid/civ626

**Published:** 2015-11-09

**Authors:** Milagritos D. Tapia, Samba O. Sow, Fadima Cheick Haidara, Fatoumata Diallo, Moussa Doumbia, Godwin C. Enwere, Gandhali Paranjape, Jacques Hervé, Enricke Bouma, Varsha Parulekar, Lionel Martellet, Julie Chaumont, Brian D. Plikaytis, Yuxiao Tang, Prasad S. Kulkarni, Katharina Hartmann, Marie-Pierre Preziosi

**Affiliations:** 1 Department of Pediatrics, Center for Vaccine Development, University of Maryland School of Medicine, Baltimore; 2 Centre pour le Développement des Vaccins, Ministère de la Santé, Bamako, Mali; 3 Meningitis Vaccine Project, PATH, Ferney-Voltaire, France; 4 DiagnoSearch Life Sciences, Mumbai, India; 5 Centers for Disease Control and Prevention, Atlanta, Georgia; 6 Meningitis Vaccine Project, PATH, Seattle, Washington; 7 Serum Institute of India Ltd, Pune; 8 Department of Pharmacovigilance and Pharmacoepidemiology, Eidgenössische Technische Hochschule, Zürich; 9 Meningitis Vaccine Project, Department of Immunization, Vaccines and Biologicals, World Health Organization, Geneva, Switzerland

**Keywords:** MenAfriVac, vaccine safety, randomized clinical trial, active control, Africa

## Abstract

***Background.*** A safe, affordable, and highly immunogenic meningococcal A conjugate vaccine (PsA-TT, MenAfriVac) was developed to control epidemic group A meningitis in Africa. Documentation of the safety specifications of the PsA-TT vaccine was warranted, with sufficient exposure to detect potential rare vaccine-related adverse reactions.

***Methods.*** This phase 3, double-blind, randomized, active controlled clinical study was designed to evaluate the safety—primarily vaccine-related serious adverse events (SAEs)—up to 3 months after administration of a single dose of the PsA-TT vaccine to subjects aged 1–29 years in Mali. Safety outcomes were also compared to those following a single dose of a licensed meningococcal ACWY polysaccharide vaccine (PsACWY).

***Results.*** No vaccine-related SAEs occurred during the 3 months of follow-up of 4004 subjects vaccinated with a single dose of PsA-TT. When compared to PsACWY (1996 subjects), tenderness at the injection site appeared to be more frequent in the PsA-TT group. However, rates of local induration, systemic reactions, adverse events (AEs), and SAEs were similar in both groups, and unsolicited AEs and SAEs were all unrelated to the study vaccines.

***Conclusions.*** The study confirmed on a large scale the excellent safety profile of a single dose of PsA-TT when administered to its entire target population of 1–29 years of age.

***Clinical Trials Registration.*** PACTR ATMR201003000191317.

Controlling group A meningococcal disease is a public health priority. Every 10–12 years, the region of sub-Saharan Africa extending from Senegal to Ethiopia, known as the meningitis belt, experiences major epidemics of meningococcal meningitis [1]. These epidemics have been most commonly caused by group A *Neisseria meningitidis*.

Since 2001, the Meningitis Vaccine Project, a partnership between the World Health Organization (WHO) and PATH, funded by the Bill & Melinda Gates Foundation, has aimed to eliminate group A meningococcal disease through the development of an affordable group A meningococcal conjugate vaccine that induces immunologic memory and a long-lasting immune response [2].

This effort led to the licensure, followed by WHO prequalification, of PsA-TT (MenAfriVac, Serum Institute of India, Ltd) in June 2010 after 4 clinical studies (phase 1, 2, and 2/3) that included a total of 1915 subjects, of whom 1126 from age 1 to 34 years had received at least 1 dose of PsA-TT, and which demonstrated its safety and superior immune response compared with the group A–containing polysaccharide vaccine [3–8]. Two additional clinical studies (phase 3), a lot consistency study in India and a large safety study in Africa, were ongoing at the time of prequalification and submitted to regulatory authorities as part of the early postlicensure requirements.

Here we present the results of the large phase 3 safety trial conducted in 2010 in Bamako, Mali, to collect additional data on the safety profile of PsA-TT in a randomized controlled setting [9–12], while allowing sufficient exposure to detect potential rare vaccine-related adverse reactions, where “rare” is conventionally defined as ≤1 per 1000 vaccinees [13].

## METHODS

### Study Design and Oversight

This phase 3, double-blind, randomized, active controlled clinical study (PsA-TT-006) was designed to evaluate the safety of a single intramuscular injection of the study vaccine up to 3 months after vaccination, in healthy residents of the study area; it was intended to increase the safety database and to further document the safety specifications of PsA-TT. The study was performed in children, adolescents, and adults aged 1–29 years, recruited at the Center for Vaccine Development in Bamako, Mali. Six thousand eligible subjects were randomized in a 2:1 ratio to receive either the study vaccine (PsA-TT, MenAfriVac, Serum Institute of India; group 1, n = 4004) or the reference vaccine (PsACWY, Mencevax ACWY, GlaxoSmithKline; group 2, n = 1996). The sample size was stratified into 3 age groups (children 1–10 years, adolescents 11–17 years, and adults 18–29 years of age) to ensure a balanced safety information across the target population of 1–29 years of age [14]. Each participant made 1 vaccination visit and 3 follow-up visits (at 4 days, 28 days, and 84 days after vaccination). Only the staff members at the study sites who were directly responsible for preparing the vaccines were aware of group assignments; the subjects and their families, other site staff members, investigators, and sponsors were blinded and unaware of group assignments throughout the study period.

The main criteria for exclusion were a history of allergic disease or known hypersensitivity to any component of the 2 study vaccines and/or following administration of vaccines included in the local program of immunization; administration of any other vaccine within 30 days prior to administration of study vaccines or planned vaccination during the first 28 days after the study vaccination; or pregnancy or lactation (prelicensing vaccine trials did at that time not usually include pregnant and lactating women). A negative pregnancy test was required before vaccination for all women of childbearing potential (ie, postmenarcheal or married women). The study was conducted in accordance with the study protocol and was designed and conducted in accordance with the Good Clinical Practice guidelines established by the International Conference on Harmonisation and with the Declaration of Helsinki. The participating community approved the study, and written informed consent was obtained before enrollment from all subjects between 18 and 29 years of age and from all parents or guardians of subjects <18 years of age. In addition, written informed assent was obtained from subjects 10–17 years of age as appropriate within the participating community. The study was approved by the PATH and WHO ethics committees, as well as by the ethics committee and the national regulatory authority in Mali.

### Vaccines

A reconstituted dose of PsA-TT (0.5 mL) contained 10 µg of group A polysaccharide conjugated to 10–33 µg of tetanus toxoid, 2.85 mg of mannitol, 0.72 mg of sucrose, 0.3 mg aluminium(III) ion as aluminum phosphate as adjuvant, TRIS buffer, 0.01% thiomersal (preservative), and 0.9% sodium chloride, and water for injection.

A reconstituted dose of reference vaccine, PsACWY (0.5 mL), contained 50 µg of purified polysaccharide from each of the *N. meningitidis* groups A, C, Y, and W, sucrose, trometamol, sodium chloride, phenol, and sterile water for injection.

The vaccines were injected intramuscularly, in the right thigh for children <2 years of age, and in the right deltoid for children aged ≥2 years, adolescents, and adults.

### Safety Evaluation

Subjects were observed for 30 minutes after vaccination to record and treat immediate reactions. Subjects were monitored for local and systemic postimmunization reactions during daily home visits by nurses and doctors using diaries for 4 days, unsolicited adverse events (AEs) were assessed for 1 month, and serious adverse events (SAEs) were assessed throughout the course of the study for 3 months. Subjects (or their parents or guardians) were asked about tenderness and induration at the injection site; fever, vomiting, and diarrhea (for all subjects); lethargy, irritability, and loss of appetite (for subjects between 1 and 10 years of age); and headache, fatigue, myalgia, and arthralgia (for subjects >10 years of age). Solicited reactions within 4 days after vaccination were presumed to be vaccine related. Assessment of causality in the case of unsolicited AEs was performed by the study investigators based on clinical judgment. An independent data and safety monitoring board was established.

### Statistical Analysis

The primary objective of this study was to evaluate the safety up to 3 months (84 days) after a single dose of PsA-TT, in terms of vaccine-related SAEs. The primary analysis on the primary endpoint was descriptive.

The safety of a single dose of PsA-TT was compared to that of 1 dose of PsACWY, in terms of local and systemic reactions, AEs, and SAEs. Differences between the 2 vaccine groups were tested using a Cochran–Mantel–Haenszel test adjusting for age group for percentage of subjects with at least 1 postimmunization reaction (local or systemic), percentage of subjects reporting the presence of each reactogenicity parameter (however, for lethargy, irritability, and loss of appetite in those aged 1–10 years, the comparison was made by Fisher exact test at a 2-sided significance level of α = .05), and percentage of subjects with at least 1 AE or SAE. Because lethargy, irritability, and loss of appetite were only collected in the age group of 1–10 years, Fisher exact test was used to compare the 2 vaccine groups for percentage of subjects with at least 1 of these 3 postimmunization reactions.

All safety analyses were carried out on the intention-to-treat dataset. Data were analyzed with SAS software, version 9.1.3. A 2-sided significance level of .05 was used for testing. Calculations of the sample size, required to assess the primary objective with sufficient power, were based on the probability of observing a rare AE as derived by a Poisson approximation [15]. When the incidence rate (R0) of a particular AE is sufficiently low and a sample size is sufficiently large, the probability of observing an AE can be approximated by that of a Poisson distribution with parameter λ = nR0. If no vaccine-related SAE was observed in the estimated 4000 participants who were supposed to receive a single dose of the PsA-TT vaccine, one can conclude with a 95% confidence that the incidence of the vaccine-related SAE for PsA-TT vaccine is <1 in 1333 vaccinated individuals.

## RESULTS

### Study Population

**Figure 1.**
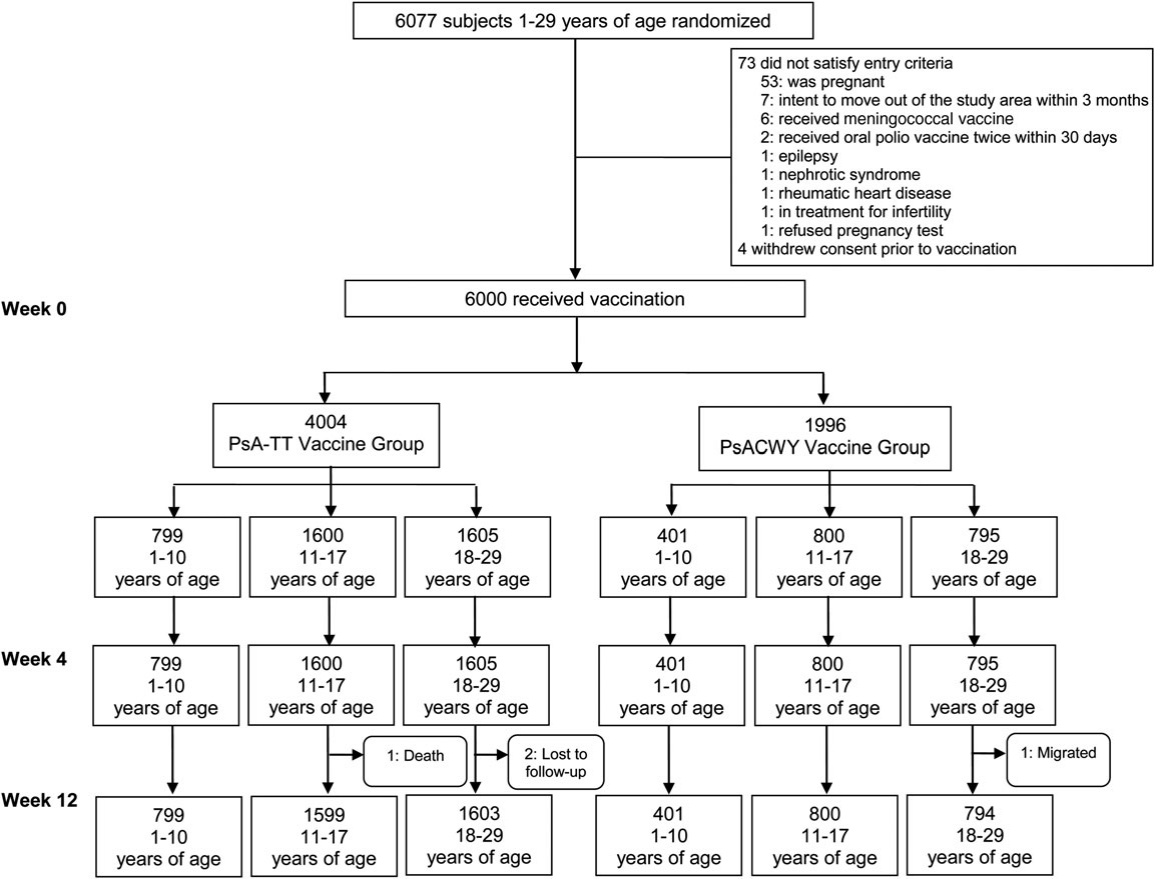
Study population. Abbreviations: PsACWY, group A, C, W, Y meningococcal polysaccharide vaccine; PsA-TT, group A meningococcal polysaccharide-tetanus toxoid conjugate vaccine.

Of the 6077 randomized subjects, 6000 were vaccinated between 22 February and 9 October 2010: 4004 in the PsA-TT group and 1996 in the PsACWY group (Figure 1). All vaccinated subjects were included in the analyses. The duration of study participation for each subject was 3 months: all subjects completed the initial vaccine period of 28 days, and all but 4 subjects completed the study with an additional 56 days of follow-up (Figure 1). Demographic and clinical characteristics of the subjects are summarized in Table 1.

**Table 1.** Age, Sex, Height, and Weight of Study Subjects

Age Group, y	Vaccine Group	No.	Median Age, Years (Min–Max)	Female, No. (%)	Median Height, cm (Min–Max)	Median Weight, kg (Min–Max)
1–10	PsA-TT	799	8 (1–10)	396 (49.6)	125.5 (70.0–159.2)	22.0 (7.0–59.0)
	PsACWY	401	7 (1–10)	187 (46.6)	124.0 (70.0–172.0)	22.0 (7.0–55.0)
11–17	PsA-TT	1600	14 (11–17)	737 (46.1)	156.5 (122.0–193.0)*	47.0 (20.0–94.5)**
	PsACWY	800	14 (11–17)	368 (46.0)	155.0 (128.0–183.4)*	45.0 (23.0–90.0)**
18–29	PsA-TT	1605	21 (18–29)	542 (33.8)	167.2 (145.1–195.0)	61.0 (36.0–129.0)
	PsACWY	795	21 (18–29)	252 (31.7)	167.3 (137.0–199.0)	61.0 (36.5–103.0)
Total	PsA-TT	4004	16 (1–29)	1675 (41.8)	159.0 (70.0–195.0)	51.0 (7.0–129.0)
	PsACWY	1996	16 (1–29)	807 (40.4)	157.6 (70.0–199.0)	50.0 (7.0–103.0)

Abbreviations: PsACWY, group A, C, W, Y meningococcal polysaccharide vaccine; PsA-TT, group A meningococcal polysaccharide-tetanus toxoid conjugate vaccine.

* *P* = .0093 for the comparison of PsACWY vs PsA-TT in the age group 11–17 years using analysis of variance (ANOVA).

** *P* = .0204 for the comparison of PsACWY vs PsA-TT in the age group 11–17 years (ANOVA).

### Safety

#### Primary Endpoint

No vaccine-related SAE occurred during the 3 months of follow-up. Therefore, no further analyses were required on the primary criterion.

#### Secondary Endpoints

Postimmunization reactions and AEs are shown in Table 2. No allergic or anaphylactic reaction occurred immediately after immunization. Rates of systemic reactions during the first 4 days after immunization, rates of AEs during the first 28 days after immunization, and SAEs within 84 days after immunization were similar among vaccine groups.

**Table 2.** Overall Vaccine Safety Profile

			Local Reaction		Systemic Reaction^a^		Adverse Event		Serious Adverse Event	
			Within 4 d Postimmunization		Within 4 d Postimmunization		Within 28 d Postimmunization		Within 84 d Postimmunization	
Age Group, y	Vaccine Group	No.	No.	% (95% CI)	No.	% (95% CI)	No.	% 95% CI)	No.	% (95% CI)
1–10	PsA-TT	799	72	9.0 (7.1–11.2)	11	1.4 (.7–2.4)	100	12.5 (10.3–15.0)	7	0.9 (.4–1.8)
	PsACWY	401	16	4.0 (2.3–6.4)	5	1.2 (.4–2.9)	61	15.2 (11.8–19.1)	3	0.7 (.2–2.2)
11–17	PsA-TT	1600	189	11.8 (10.3–13.5)	32	2.0 (1.4–2.8)	119	7.4 (6.2–8.8)	11	0.7 (.3–1.2)
	PsACWY	800	35	4.4 (3.1–6.0)	19	2.4 (1.4–3.7)	79	9.9 (7.9–12.2)	5	0.6 (.2–1.5)
18–29	PsA-TT	1605	265	16.5 (14.7–18.4)	73	4.5 (3.6–5.7)	187	11.7 (10.1–13.3)	16	1.0 (.6–1.6)
	PsACWY	795	35	4.4 (3.1–6.1)	40	5.0 (3.6–6.8)	92	11.6 (9.4–14.0)	6	0.8 (.3–1.6)
Total	PsA-TT	4004	526	13.1^b^ (12.1–14.2)	116	2.9 (2.4–3.5)	406	10.1 (9.2–11.1)	34	0.8 (.6–1.2)
	PsACWY	1996	86	4.3^b^ (3.5–5.3)	64	3.2 (2.5–4.1)	232	11.6 (10.2–13.1)	14	0.7 (.4–1.2)

Abbreviations: CI, confidence interval; PsACWY, group A, C, W, Y meningococcal polysaccharide vaccine; PsA-TT, group A meningococcal polysaccharide–tetanus toxoid conjugate vaccine.

^a^ Subjects reporting at least 1 of fever, vomiting, and diarrhea: age group 1–10 years, 1.0% of subjects (95% CI, .4%–2.0%; n/N = 8/799) in the PsA-TT group and 0.7% of subjects (95% CI, .2%–2.2%; n/N = 3/401) in the PsACWY group; 11–17 years, 0.3% of subjects (95% CI, .1%–.7%; n/N = 5/1600) in PsA-TT and 0.4% of subjects (95% CI, .1%–1.1%; n/N = 3/800) in PsACWY; and 18–29 years, 0.6% of subjects (95% CI, .3%–1.1%; n/N = 9/1605) in PsA-TT and 0.6% of subjects (95% CI, .2%–1.5%; n/N = 5/795) in PsACWY. Subjects reporting at least 1 of lethargy, irritability, and loss of appetite: age group 1–10 years, 0.8% of subjects (95% CI, .3%–1.6%; n/N = 6/799) in the PsA-TT group and 0.5% of subjects (95% CI, .1%–1.8%; n/N = 2/401) in the PsACWY group. Subjects reporting at least 1 of headache, fatigue, myalgia, and arthralgia: age group 11–17 years, 1.8% of subjects (95% CI, 1.2%–2.6%; n/N = 29/1600) in PsA-TT and 2.1% of subjects (95% CI, 1.2%–3.4%; n/N = 17/800) in PsACWY; 18–29 years, 4.2% of subjects (95% CI, 3.3%–5.3%; n/N = 68/1605) in PsA-TT and 4.7% of subjects (95% CI, 3.3%–6.4%; n/N = 37/795) in PsACWY.

^b^ *P* < .0001 for the comparison of PsACWY vs PsA-TT using Cochran–Mantel–Haenszel test adjusting for age group. The difference was due to more tenderness reported in the PsA-TT than PsACWY group.

However, the rate of local reactions was 3 times as high in the PsA-TT group as in the PsACWY group (13.1% vs 4.3%; *P* < .0001). All but 2 mild indurations were injection-site tenderness, with a between-group rate difference increasing with age of the subjects. Intensity of tenderness was rated 1 (mild pain to touch) for all subjects in the PsACWY group and for most of the subjects in the PsA-TT group. In the PsA-TT group, tenderness intensity rated 2 (significant pain to touch) was reported as follows: 0 children, 7 adolescents (3.7% of 189 adolescents who reported tenderness), and 21 adults (8.0% of 264 adults who reported tenderness). Only 1 adult reported a tenderness of intensity 3 (significant pain on moving the limb).

Rates of systemic reactions were low (2.9% in PsA-TT vs 3.2% in PsACWY) and increased with age of the subjects: 1.4% vs 1.2% in children; 2.0% vs 2.4% in adolescents, and 4.5% vs 5.0% in adults in the PsA-TT vs PsACWY groups, respectively. Headache, fatigue, myalgia, and arthralgia (solicited only among adolescents and adults) were the most frequently reported systemic postimmunization reactions, with 3.0% vs 3.4% of the subjects in the PsA-TT vs PsACWY group, respectively, reporting at least 1 of these 4 reactions. Headache was the most frequently reported reaction: 2.3% in PsA-TT vs 2.8% in PsACWY, twice as frequent in adults than in adolescent subjects. Fatigue was reported more frequently in the PsACWY group (1.1% vs 0.5% in the PsA-TT group; *P* = .0160). Overall, local and systemic postimmunization reactions were mild, transient, and resolved without sequelae.

A total of 10.1% subjects in the PsA-TT group and 11.6% of subjects in the PsACWY group reported at least 1 unsolicited AE. Commonly reported AEs were infections and infestations according to system organ class (SOC), mainly malaria, upper respiratory tract infections, schistosomiasis, and rhinitis. Adverse events were transient and resolved without sequelae, and no vaccine-related unsolicited AEs were recorded.

Within 3 months after vaccination, 48 subjects—34 subjects (0.8%) in the PsA-TT group and 14 (0.7%) in the PsACWY group—reported experiencing 50 serious AEs, all of which were vaccine unrelated (Table 3). One adolescent had vascular disorder according to SOC (severe malignant hypertension) and died 57 days after vaccination with PsA-TT; the event was unrelated to the study vaccine. Twenty-nine subjects (0.7%) in the PsA-TT group and 14 (0.7%) in the PsACWY group reported a total of 45 SAEs that were infections and infestations according to SOC, mainly malaria (34 cases). The remaining 4 SAEs were 2 cases of injury, poisoning, and procedural complications (multiple injuries, forearm fracture), and 1 case each of gastrointestinal disorders (appendicitis) and surgical and medical procedures (induced abortion) and were reported in 4 subjects in the PsA-TT group. Most of the cases of SAEs were severe (43/50) or moderate (7/50), but all, except for the fatal case, recovered without sequelae. The overall comparison adjusted for age groups showed no statistically significant difference in the percentage of subjects with at least 1 SAE between the 2 vaccine groups with respect to SAEs after vaccination. There was no difference when comparing the rates within each follow-up period of before or after the first 28 days postimmunization.

**Table 3.** Summary of Subjects With Severe Adverse Events by Primary System Organ Class

Age Group, y	Vaccine Group		Primary System Organ Class					
			Infections and Infestations^a^	Gastrointestinal Disorders	Injury, Poisoning, and Procedural Complications	Surgical and Medical Procedures	Vascular Disorder^b^	All
		No.	No. (%)	No. (%)	No. (%)	No. (%)	No. (%)	No. (%)
1–10	PsA-TT	799	6 (0.8)	0 (0.0)	1 (0.1)	0 (0.0)	0 (0.0)	7 (0.9)
	PsACWY	401	3 (0.7)	0 (0.0)	0 (0.0)	0 (0.0)	0 (0.0)	3 (0.7)
11–17	PsA-TT	1600	9 (0.6)	0 (0.0)	0 (0.0)	1 (0.1)	1 (0.1)	11 (0.7)
	PsACWY	800	5 (0.6)	0 (0.0)	0 (0.0)	0 (0.0)	0 (0.0)	5 (0.6)
18–29	PsA-TT	1605	14 (0.9)	1 (0.1)	1 (0.1)	0 (0.0)	0 (0.0)	16 (1.0)
	PsACWY	795	6 (0.8)	0 (0.0)	0 (0.0)	0 (0.0)	0 (0.0)	6 (0.8)
Total	PsA-TT	4004	29 (0.7)	1 (0.0)	2 (0.0)	1 (0.0)	1 (0.0)	34 (0.8)
	PsACWY	1996	14 (0.7)	0 (0.0)	0 (0.0)	0 (0.0)	0 (0.0)	14 (0.7)

Abbreviations: PsACWY, group A, C, W, Y meningococcal polysaccharide vaccine; PsA-TT, group A meningococcal polysaccharide–tetanus toxoid conjugate vaccine.

^a^ Only 2 subjects reported >1 SAE: 1 subject aged 1–10 years in the PsA-TT group and the other aged 1–10 years in the PsACWY group reported 2 SAEs that were infections and infestations.

^b^ One SAE that was vascular disorder (malignant hypertension) resulted in death of a subject aged 11–17 years in the PsA-TT group.

For detailed data on solicited local and systemic reactions, AEs, and SAEs, see the Supplementary Tables.

#### Pregnancies

Within the study period, 4 pregnancies were reported 1–2 months after vaccination among 3 adults and 1 adolescent. Three subjects had normal live-born deliveries 8–9 months after vaccination, and 1 had a voluntary termination of pregnancy at 2 months after vaccination.

## DISCUSSION

This study increased the safety database while further documenting the safety specifications of PsA-TT in a randomized controlled setting. No vaccine-related SAE occurred during the 84-day follow-up of 4004 subjects vaccinated with a single dose of PsA-TT, which supports that PsA-TT vaccine is safe. Indeed, rare events are conventionally defined as those whose frequency is ≤1 per 1333 vaccinated individuals [13, 16]. Rare events could be ruled out, as no vaccine-related SAEs were observed among the 4004 subjects vaccinated with PsA-TT.

When compared to the licensed PsACWY vaccine, local reactogenicity appeared to be more frequent in the PsA-TT vaccine group, with a high number of instances of tenderness at the injection site (3 times as frequent). This was also seen in previous studies [4–8] and can be explained by the presence of TT as a carrier protein and aluminum as an adjuvant. Occurrence and intensity of tenderness increased with the age of the vaccinated subjects and were more frequent among adults. However, the rates of systemic reactions, AEs, and SAEs were similar in both vaccine groups. All AEs, whether they were serious or not, were unrelated to the study vaccines and consisted mainly of infection and infestations, whose distribution was consistent with the pattern of the age-related morbidity in Mali.

In conclusion, this study confirmed the excellent safety profile of 1 dose of MenAfriVac when administered to its entire target population of 1–29 years of age, thus supporting the prospects of large vaccine deployment in countries of the African meningitis belt.

## Supplementary Data

Supplementary materials are available at *Clinical Infectious Diseases* online (http://cid.oxfordjournals.org). Supplementary materials consist of data provided by the author that are published to benefit the reader. The posted materials are not copyedited. The contents of all supplementary data are the sole responsibility of the authors. Questions or messages regarding errors should be addressed to the author.

Supplementary Data
