# Supplementary material for: A Phase 3, Double-Blind, Randomized, Active Controlled Study to Evaluate the Safety of MenAfriVac in Healthy Malians
Source: Clin Infect Dis. 2015 Nov 9;61(Suppl 5):S507–13. doi: 10.1093/cid/civ626 (PMC4639507; doi:10.1093/cid/civ626)
Supplement: Supplementary Data [file supp_61_suppl-5_S507__index.html]

Supplementary Data 

# A Phase 3, Double-Blind, Randomized, Active Controlled Study to Evaluate the Safety of MenAfriVac in Healthy Malians

## Supplementary Data

Supplementary Data

- Supplementary Data - Docx file
