# Supplementary material for: A Phase 3, Double-Blind, Randomized, Active Controlled Study to Evaluate the Safety of MenAfriVac in Healthy Malians
Source: Clin Infect Dis. 2015 Nov 9;61(Suppl 5):S507–13. doi: 10.1093/cid/civ626 (PMC4639507; doi:10.1093/cid/civ626)
Supplement: Supplementary Data [file supp_civ626_civ626supp.docx]

| **Supplementary Table 1. Summary of subjects with local post-immunization reactions** | | | | | | | |  |
| --- | --- | --- | --- | --- | --- | --- | --- | --- |
| **Age group** | **Vaccine group** | | **Tenderness** | | | **Induration** | | |
|  |  | **N** | **n** | **%** | **(95% CI)** | **n** | **%** | **(95% CI)** |
| 1-10 yrs | PsA-TT | 799 | 72 | 9.0 | (7.1-11.2) | 0 | 0.0 | (0.0-0.5) |
|  | PsACWY | 401 | 16 | 4.0 | (2.3-6.4) | 0 | 0.0 | (0.0-0.9) |
| 11-17 yrs | PsA-TT | 1600 | 189 | 11.8 | (10.3-13.5) | 0 | 0.0 | (0.0-0.2) |
|  | PsACWY | 800 | 35 | 4.4 | (3.1-6.0) | 0 | 0.0 | (0.0-0.5) |
| 18-29 yrs | PsA-TT | 1605 | 264 | 16.4 | (14.7-18.4) | 2 | 0.1 | (0.0-0.4) |
|  | PsACWY | 795 | 35 | 4.4 | (3.1-6.1) | 0 | 0.0 | (0.0-0.5) |
| Total | PsA-TT | 4004 | 525 | 13.1* | (12.1-14.2) | 2 | 0.0 | (0.0-0.2) |
|  | PsACWY | 1996 | 86 | 4.3* | (3.5-5.3) | 0 | 0.0 | (0.0-0.2) |
| * P < 0.0001 for the comparison of PsACWY vs. PsA-TT using CMH test adjusting for age group. | | | | | | |  |  |

| **Supplementary Table 2. Summary of subjects with systemic post-immunization reactions** | | | | | | | | | | | |  |  |  |  |  |  |  |  |  |
| --- | --- | --- | --- | --- | --- | --- | --- | --- | --- | --- | --- | --- | --- | --- | --- | --- | --- | --- | --- | --- |
| **Age group** | **Vaccine group** | | **Fever** | | | **Vomiting** | | | **Diarrhea** | | | **Lethargy** | | | **Irritability** | | | **Loss of appetite** | | |
|  |  | **N** | **n** | **%** | **(95% CI)** | **n** | **%** | **(95% CI)** | **n** | **%** | **(95% CI)** | **n** | **%** | **(95% CI)** | **n** | **%** | **(95% CI)** | **n** | **%** | **(95% CI)** |
| 1-10 yrs | PsA-TT | 799 | 2 | 0.3 | (0.0-0.9) | 2 | 0.3 | (0.0-0.9) | 4 | 0.5 | (0.1-1.3) | 3 | 0.4 | (0.1-1.1) | 1 | 0.1 | (0.0-0.7) | 4 | 0.5 | (0.1-1.3) |
|  | PsACWY | 401 | 1 | 0.2 | (0.0-1.4) | 0 | 0.0 | (0.0-0.9) | 2 | 0.5 | (0.1-1.8) | 1 | 0.2 | (0.0-1.4) | 1 | 0.2 | (0.0-1.4) | 2 | 0.5 | (0.1-1.8) |
| 11-17 yrs | PsA-TT | 1600 | 1 | 0.1 | (0.0-0.3) | 2 | 0.1 | (0.0-0.5) | 2 | 0.1 | (0.0-0.5) |  |  |  |  |  |  |  |  |  |
|  | PsACWY | 800 | 0 | 0.0 | (0.0-0.5) | 2 | 0.3 | (0.0-0.9) | 1 | 0.1 | (0.0-0.7) |  |  |  |  |  |  |  |  |  |
| 18-29 yrs | PsA-TT | 1605 | 0 | 0.0 | (0.0-0.2) | 2 | 0.1 | (0.0-0.4) | 8 | 0.5 | (0.2-1.0) |  |  |  |  |  |  |  |  |  |
|  | PsACWY | 795 | 0 | 0.0 | (0.0-0.5) | 3 | 0.4 | (0.1-1.1) | 2 | 0.3 | (0.0-0.9) |  |  |  |  |  |  |  |  |  |
| Total | PsA-TT | 4004 | 3 | 0.1 | (0.0-0.2) | 6 | 0.1 | (0.1-0.3) | 14 | 0.3 | (0.2-0.6) | 3 | 0.4 | (0.1-1.1) | 1 | 0.1 | (0.0-0.7) | 4 | 0.5 | (0.1-1.3) |
|  | PsACWY | 1996 | 1 | 0.1 | (0.0-0.3) | 5 | 0.3 | (0.1-0.6) | 5 | 0.3 | (0.1-0.6) | 1 | 0.2 | (0.0-1.4) | 1 | 0.2 | (0.0-1.4) | 2 | 0.5 | (0.1-1.8) |
|  |  |  | **Headache** | | | **Fatigue** | | | **Myalgia** | | | **Arthralgia** | | |  |  |  |  |  |  |
|  |  | **N** | **n** | **%** | **(95% CI)** | **n** | **%** | **(95% CI)** | **n** | **%** | **(95% CI)** | **n** | **%** | **(95% CI)** |  |  |  |  |  |  |
| 1-10 yrs | PsA-TT |  |  |  |  |  |  |  |  |  |  |  |  |  |  |  |  |  |  |  |
|  | PsACWY |  |  |  |  |  |  |  |  |  |  |  |  |  |  |  |  |  |  |  |
| 11-17 yrs | PsA-TT | 1600 | 25 | 1.6 | (1.0-2.3) | 3 | 0.2 | (0.0-0.5) | 2 | 0.1 | (0.0-0.5) | 4 | 0.3 | (0.1-0.6) |  |  |  |  |  |  |
|  | PsACWY | 800 | 15 | 1.9 | (1.1-3.1) | 3 | 0.4 | (0.1-1.1) | 1 | 0.1 | (0.0-0.7) | 0 | 0.0 | (0.0-0.5) |  |  |  |  |  |  |
| 18-29 yrs | PsA-TT | 1605 | 50 | 3.1 | (2.3-4.1) | 12 | 0.7 | (0.4-1.3) | 13 | 0.8 | (0.4-1.4) | 10 | 0.6 | (0.3-1.1) |  |  |  |  |  |  |
|  | PsACWY | 795 | 30 | 3.8 | (2.6-5.3) | 14 | 1.8 | (1.0-2.9) | 3 | 0.4 | (0.1-1.1) | 4 | 0.5 | (0.1-1.3) |  |  |  |  |  |  |
| Total | PsA-TT | 4004 | 75 | 2.3 | (1.8-2.9) | 15 | 0.5* | (0.3-0.8) | 15 | 0.5 | (0.3-0.8) | 14 | 0.4 | (0.2-0.7) |  |  |  |  |  |  |
|  | PsACWY | 1996 | 45 | 2.8 | (2.1-3.8) | 17 | 1.1* | (0.6-1.7) | 4 | 0.3 | (0.1-0.6) | 4 | 0.3 | (0.1-0.6) |  |  |  |  |  |  |
|  | | | | | | | | | |  |  |  |  |  |  |  |  |  |  |  |

* P = 0.0160 for the comparison of PsACWY vs. PsA-TT using CMH test adjusting for age group.

**Supplementary Table 3** **Summary of subjects with AEs by primary system organ class**

| **Primary System Organ Class** | |  | **Infections and infestations** | **Injury, poisoning and procedural complications** | **Gastrointestinal disorders** | **Respiratory, thoracic and mediastinal disorders** | **Skin and subcutaneous tissue disorders** | **Nervous system disorders** | **Eye disorders** | **Resproductive, thoracic and mediastinal disorders** | **Muskuloskeletal and connective tissue disorders** | **General disorders and administration site conditions** | **Ear and labyrinth disorders** | **Metabolism and nutrition disorders** | **Renal and urinary disorders** | **ALL** |
| --- | --- | --- | --- | --- | --- | --- | --- | --- | --- | --- | --- | --- | --- | --- | --- | --- |
| **Age group**  **In years** | **Vaccine group** | **N** | **n (%)** | **n (%)** | **n (%)** | **n (%)** | **n (%)** | **n (%)** | **n (%)** | **n (%)** | **n (%)** | **n (%)** | **n (%)** | **n (%)** | **n (%)** | **n (%)** |
| 1-10 | PsA-TT | 799 | 83 (10.4) | 6 (0.8) | 4 (0.5) | 8 (1.0) | 5 (0.6) | 1 (0.1) | 1 (0.1) | 1 (0.1) | 0 (0.0) | 1 (0.1) | 0 (0.0) | 0 (0.0) | 0 (0.0) | 100 (12.5) |
|  | PsACWY | 401 | 49 (12.2) | 1 (0.2) | 0 (0.0) | 6 (1.5) | 5 (1.2) | 1 (0.2) | 2 (0.5) | 0 (0.0) | 1 (0.2) | 1 (0.2) | 0 (0.0) | 0 (0.0) | 0 (0.0) | 61 (15.2) |
| 11-17 | PsA-TT | 1600 | 92 (5.8) | 12 (0.8) | 6 (0.4) | 8 (0.5) | 3 (0.2) | 0 (0.0) | 1 (0.1) | 0 (0.0) | 0 (0.0) | 1 (0.1) | 1 (0.1) | 0 (0.0) | 0 (0.0) | 119 (7.4) |
|  | PsACWY | 800 | 57 (7.1) | 6 (0.8) | 2 (0.3) | 5 (0.6) | 2 (0.3) | 5 (0.6) | 2 (0.3) | 0 (0.0) | 0 (0.0) | 0 (0.0) | 1 (0.1) | 0 (0.0) | 0 (0.0) | 79 (9.9) |
| 18-29 | PsA-TT | 1605 | 118 (7.4) | 16 (1.0) | 25 (1.6) | 7 (0.4) | 6 (0.4) | 5 (0.3) | 4 (0.2) | 8 (0.5) | 4 (0.2) | 1 (0.1) | 0 (0.0) | 1 (0.1) | 1 (0.1) | 187 (11.7) |
|  | PsACWY | 795 | 67 (8.4) | 9 (1.1) | 11 (1.4) | 5 (0.6) | 1 (0.1) | 0 (0.0) | 0 (0.0) | 1 (0.1) | 0 (0.0) | 0 (0.0) | 0 (0.0) | 0 (0.0) | 0 (0.0) | 92 (11.6) |
| Total | PsA-TT | 4004 | 293 (7.3) | 34 (0.8) | 35 (0.9) | 23 (0.6) | 14 (0.3) | 6 (0.1) | 6 (0.1) | 9 (0.2) | 4 (0.1) | 3 (0.1) | 1 (0.0) | 1 (0.0) | 1 (0.0) | 406 (10.1) |
|  | PsACWY | 1996 | 173 (8.7) | 16 (0.8) | 13 (0.7) | 16 (0.8) | 8 (0.4) | 6 (0.3) | 4 (0.2) | 1 (0.1) | 1 (0.1) | 1 (0.1) | 1 (0.1) | 0 (0.0) | 0 (0.0) | 232 (11.6) |
| Note: Adverse events reported in this table do not include local and systemic reactions ongoing beyond 4 days post-immunization. | | | | | | |  |  |  |  |  |  |  |  |  |  |
